# Motion‐corrected brain MRI at ultralow field (64 mT)

**DOI:** 10.1002/mrm.30506

**Published:** 2025-03-28

**Authors:** Yannick Brackenier, Rui Pedro Teixeira, Lucilio Cordero‐Grande, Emil Ljungberg, Niall J. Bourke, Tomoki Arichi, Sean Deoni, Steve C. R. Williams, Joseph V. Hajnal

**Affiliations:** ^1^ Biomedical Engineering Department, School of Biomedical Engineering and Imaging Sciences King's College London London United Kingdom; ^2^ Research Department of Early Life Imaging, School of Biomedical Engineering and Imaging Sciences King's College London London United Kingdom; ^3^ Hyperfine Guilford Connecticut USA; ^4^ Biomedical Image Technologies ETSI Telecomunicación, Universidad Politécnica de Madrid and CIBER‐BNN, ISCIII Madrid Spain; ^5^ Department of Medical Radiation Physics Lund University Lund Sweden; ^6^ Department of Neuroimaging King's College London London United Kingdom; ^7^ Bill & Melinda Gates Foundation Seattle Washington USA

**Keywords:** brain, motion correction, reconstruction, ultralow field

## Abstract

**Purpose:**

The study investigates the feasibility of applying a retrospective motion‐correction technique to ultralow‐field (ULF) MRI data to improve reconstructed image quality when there is patient motion, which is likely to be a critical challenge in portable, point‐of‐care imaging.

**Theory & Methods:**

The study tests alignedSENSE, an iterative motion correction and reconstruction method with SENSE, for ULF MRI, with additional corrections to estimate and correct within‐scan phase variations. The method was applied to in vivo brain volumetric data acquired from five healthy volunteers using a 64 mT portable MRI scanner. The volunteers underwent different motion types and levels, with corrections evaluated using both visual and quantitative metrics.

**Results:**

Motion correction, particularly when within‐scan phase variations are also accounted for, showed clear improvements in image quality. Without making any assumptions about the origin of these phase variations, incorporating them into the signal model and jointly estimating with the image/motion parameters increases the data consistency. This improves the image quality and motion parameters across various levels of induced motion. Quantitative analysis confirmed that the combined motion and phase corrections outperformed conventional parallel imaging reconstruction, although extreme motion cases still pose challenges.

**Conclusion:**

The study demonstrates that alignedSENSE motion‐correction techniques can be effectively applied to ULF MRI systems. The results suggest that these techniques can substantially enhance image quality without increasing scan time, which could make ULF MRI more clinically viable for point‐of‐care deployment.

## 1 INTRODUCTION

In recent years, there has been a growing interest in MRI systems that operate at significantly lower magnetic field strengths than those utilized in conventional clinical scanning.^1^, ^2^, ^3^, ^4^, ^5^ These innovative systems prioritize clinical value, with an emphasis on portability and cost efficiency. A major advantage of these low‐field MRI systems is their reduced infrastructure requirements, which make them a promising option for expanding the accessibility of MRI technology beyond specialized radiology departments in well‐resourced settings. Ultralow field (ULF) point‐of‐care MRI systems exemplify this innovation, being specifically engineered to capture images without disrupting ongoing medical treatment and providing crucial on‐the‐spot diagnostic information to support clinical decision‐making processes.^6^

Despite these advantages, portable MRI systems face several challenges. Primarily due to their operation at low field strengths, a noticeable reduction in the signal to noise ratio (SNR) is observed. A straightforward solution to this issue is increasing the total scan time to allow signal averaging. However, prolonged scanning can cause additional issues, particularly an increased risk of patient motion occurring during the acquisition.^7^ This can severely impact image quality and potentially the applicability of ULF for clinical scanning, especially in patient cohorts that cannot conform to ideal scanning practices.^8^

Fortunately, motion correction has been extensively researched within the MRI community, yielding many effective solutions.^9^ Of high immediate relevance for ULF MRI are methods involving retrospective motion correction without the explicit addition of navigators or external motion sensors because these solutions do not increase acquisition time or add dedicated hardware. Navigator‐based approaches that do not increase scan time exist but rely on available dead time in the sequence, which might not always be guaranteed. As a popular alternative, self‐navigated trajectories modify the acquisition sampling so that motion histories can be estimated from the imaging data.

Retrospective motion correction using self‐navigated sampling usually entails estimation of motion directly from the acquired k‐space data. The motion‐estimation process can either be performed separately or together with the image reconstruction (resulting in an alternating estimation of both the image and the motion). The former is usually achieved by acquiring a low‐resolution reference (scout) image that is specifically acquired for estimating the motion, whereas the latter do not leverage such additional information but need some form of dedicated k‐space sampling trajectory (e.g., distributed and incoherent sampling order for reconstruction deblurring using encoding redundancy (DISORDER), PROPELLER) to achieve robust performance. State‐of‐the‐art implementations of the two options are alignedSENSE for scout‐free correction^10^ and scout accelerated motion estimation and reduction (SAMER) for scout‐based correction.^11^

Here we report an initial study to test the feasibility of motion correction using alignedSENSE^10^ and an optimized temporally distributed sampling scheme (DISORDER)^12^ in the ULF regime. We present a motion experiment at ULF, showcasing the applicability of motion correction using the chosen method.

## 2 THEORY

### 2.1 AlignedSENSE retrospective motion correction

Aligned SENSE addresses motion estimation *and* correction by formulating it as an inverse problem in matrix form, where a dedicated operator is used to model the within‐scan motion.^10^ For volumetric Cartesian encoding using array receiver coils, k‐space readouts are divided into temporal segments. Each segment *n* has a single motion state attributed with pose parameters $z_{n}$, which can be estimated in addition to the volumetric image (x). This results in the generalized reconstruction in matrix form

(1) $$\left(\hat{x},\hat{z_{n}}\right)=\text{argmi}n_{x,z_{n}}\sum_{n=1:N}{\left|\left|A_{n}\mathrm{FST}\left(z_{n}\right)x-y_{n}\right|\right|}_{2}^{2},$$

where S are sensitivity profiles of the coil receiver array and F is the discrete Fourier transform. For each segment n, $T\left(z_{n}\right)$ is the rigid motion operator with motion parameters $z_{n}$, $A_{n}$ the sampling mask and $y_{n}$ the measured data for all coil elements. The structure and sizes of the operators in Equation 1 can be found in Ref. 10, together with the optimization specifications. After solving the optimization problem in Equation 1, both the motion‐corrected image ($\hat{x}$) and the motion estimates ($\hat{z_{n}}$) are obtained.

### 2.2 Per motion state spatial phase correction

Apart from patient motion, other scan nonidealities can occur throughout a scanning session. Not including these nonidealities in the signal model (Equation 1) can result in additional image artifacts. A common nonideality is within‐scan phase variation.^13^, ^14^ Previous work at high‐field systems has focused on pose‐dependent phase variations arising from the action of the polarizing magnetic field (*B*_0_) on the subject. When these variations have suitable spatial smoothness, they can be robustly estimated along with the rigid motion parameters $\hat{z_{n}}.$ ^15^ With spatial phase variations, ***p***, represented using a set of spatially low‐order basis functions L with coefficients c (such that p=Lc), the alignedSENSE forward model and reconstruction can be augmented to:

(2) $$\left(\hat{x},\hat{z_{n}},\hat{c_{n}}\right)=\underset{x,z_{n},c_{n}}{\text{argmin}}\sum_{n}{\left|\left|A_{n}\mathrm{FST}\left(z_{n}\right)P\left(c_{n}\right)x-y_{n}\right|\right|}_{2}^{2},$$

where $P\left(c_{n}\right)$ is the spatially varying phase $e^{2\pi iLc_{n}}$ of the object for segment *n*. With this formulation, image, motion, and phase variations are jointly estimated by performing an alternating optimization.^16^

Although susceptibility‐related *B*_0_ variations are expected to have minimal effect at ULF MRI,^17^ other sources of phase variations can still affect ULF MRI (e.g., eddy‐current effects, and the movement of an object in a spatially varying static *B*_0_ field). Without making any assumptions about the origin of these fields, incorporating them into the signal model and jointly estimating them could help increase the data consistency and improve the image quality.

### 2.3 Sampling patterns optimized for motion correction

Although Equations 1 and 2 can in theory be used for any sampling trajectory, robust performance of the joint estimation requires the use of self‐navigated sampling schemes. Here, we use the DISORDER sampling scheme,^12^ which consists of the repeated acquisition of shots for which each shot collects samples spanning across k‐space.^12^ After all shots are acquired, each sample on the (potentially undersampled) Cartesian k‐space grid has been sampled exactly once. To achieve this, the phase‐encoding grid is divided into tiles for which each shot acquires a single sample from every tile. In the original DISORDER paper, a “zigzag” ordering was used to traverse the different tiles, and the selection of samples within each tile is random. By default, a segment, which determines the portion of k‐space information available for each of the motion states to be estimated, consists of a single shot.

## 3 METHODS

### 3.1 In vivo data acquisition

Volumetric Cartesian acquisitions were acquired on five adult healthy volunteer (HV) subjects on a 64 mT scanner (Swoop, Hyperfine, Guilford, CT) with an inbuilt eight‐element receiver coil array. Volunteers gave written consent, and data was acquired as part of an ethically approved study. The base sequence used in this study was a turbo spin echo (TSE) with 1.96 × 1.96 × 3.92 mm^3^ resolution, TE/TR = 4.97/1000 ms, echo spacing = 4.97 ms, nonselective 90° excitation, constant nonselective 180° refocusing, FOV = 220 × 220 × 220 mm^3^, no acceleration, and acquisition time = 6 min 32 s. Echo train lengths of 32 readouts were acquired per shot. Following the vendor's acquisition setup, the 32 readouts consist of two sets of 16 readouts, which are acquired in an interleaved manner. The PE sampling pattern for the readouts within a set is identical between both sets. These repeats are referred to as *echoes* in the context of this study.

For each HV, three inversion‐recovery TSE sequences were acquired, each with an inversion time (TI) of 270 ms. For the first acquisitions, the HVs were instructed to remain still (referred to as *minimal motion*). For the next two acquisitions, the HVs were instructed to move to distinct new poses every 30 s. This was repeated twice to create different levels of motion (referred to as *deliberate motion 1* and *deliberate motion 2*, where a higher level of motion was instructed for the latter compared to the former). Only the deliberate motion 1 experiment was acquired in HV1.

For all acquisitions, an inferior–superior frequency encoding (readout) was used. This allows the neck area to be excluded from the rigid motion estimation, which is unlikely to conform with the rigid motion assumption of the brain. For each scan, coil array sensitivity profiles were estimated from a down‐sampled version ($4\times 4\times 4\;mm^{3}$) of the actual scan using a custom implementation of the ESPIRiT algorithm.^18^ An example of the coil sensitivities can be found in Supporting Information Figure S1.

### 3.2 Sampling

**FIGURE 1:**
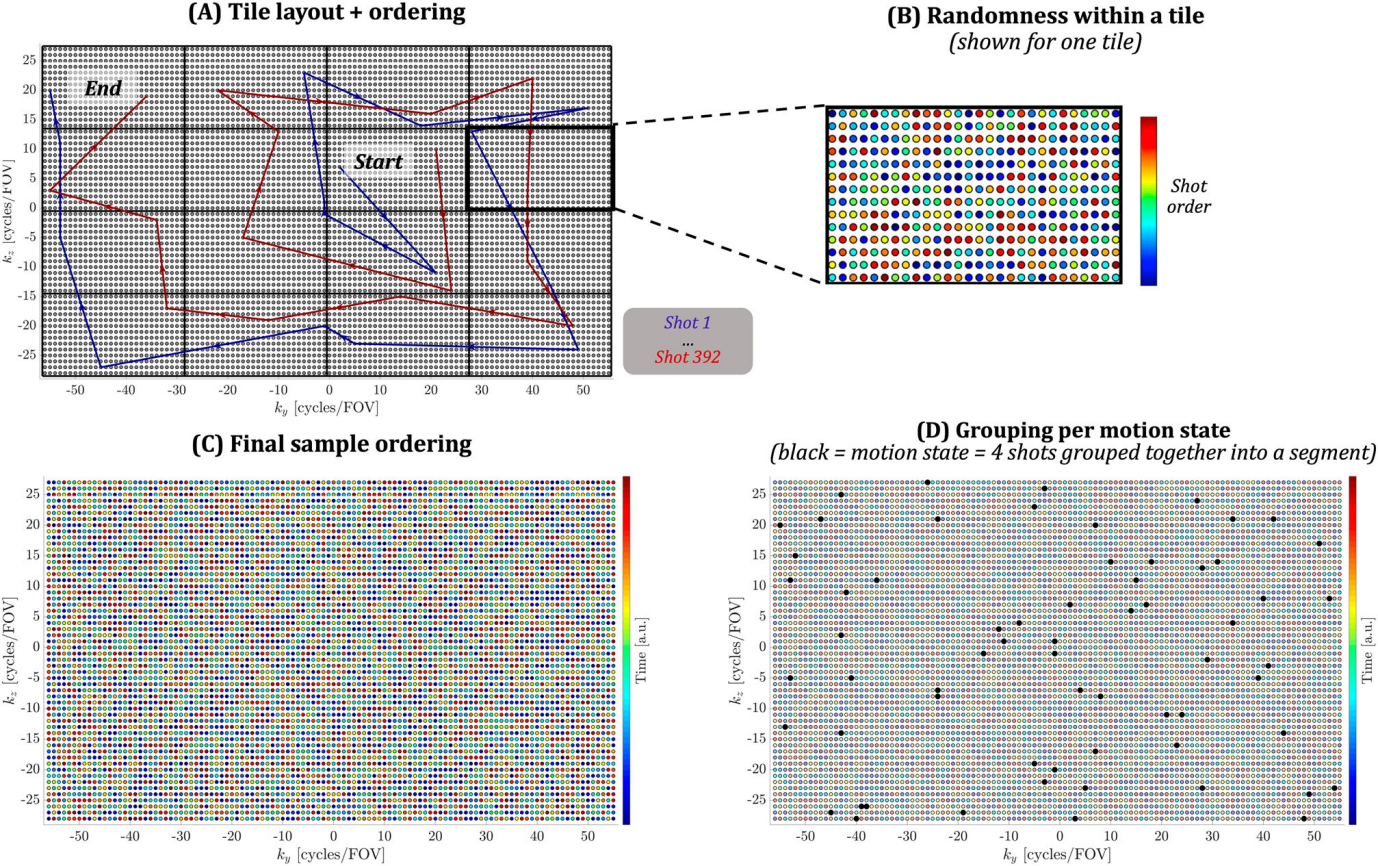
Construction of the proposed DISORDER sampling. (A) The phase encoding (PE) grid is divided into different tiles. For each shot, a single sample is extracted from each tile, where the tiles are traversed in a spiral order (arrows), shown for the first and last shot. (B) Across shots, the coordinate extracted from each tile is chosen in a random order. The random order is different for each tile, yielding the (C) final sample layout ordering in the PE plane. (D) In the context of this work, four shots are grouped together as a segment, for which motion is estimated. Samples belonging to a single segment (four shots) are shown in black. DISORDER, distributed and incoherent sampling order for reconstruction deblurring using encoding redundancy; PE, phase encoding.

The conventional DISORDER tile layout is zigzag; within each segment, the tiles are traversed from one side of k‐space to the other side in a zigzag manner. As with other sampling schemes, the zigzag tile ordering introduces specific signal weighting at the center of k‐space. Although this can be used to generate the desired contrast (e.g., MPRAGE^19^), it usually requires or leverages a long sequence of echoes per traverse. In this study, however, we used a TSE sequence with a limited number of echoes (see below). This was done to ensure that as much signal as possible from each contributing readout was leveraged, at the cost of reduced T_2_ weighting and hence contrast. To further maximize the capture of as much signal as possible within each readout, the tiles are traversed in a center‐first spiral order, as shown in Figure 1. A 4 × 4 tiling pattern was employed, resulting in a total of 392 shots per acquisition with a shot consisting of 2 × 16 readouts and duration TA_shot_ = 2 × 80 ms (considering both echoes).

### 3.3 Image reconstruction

For each acquisition, the two echoes were reconstructed separately. Three different methods were compared:

1. Uncorrected, corresponding to conventional SENSE,^20^
2. Motion corrected, corresponding to alignedSENSE,
3. Motion + phase corrected, corresponding to the reconstruction in Ref. 16.

Estimated motion and phase parameters can be obtained from each echo; however, because the echoes are acquired in an interleaved manner, those parameters can also be shared between them. From initial testing (not shown), it was found that using the estimated parameters from the first echo alone was more effective. As a result, echo 2 is reconstructed using the estimated parameters from echo 1. For robustness, motion was estimated at a slightly lower temporal resolution than provided by the sampling pattern itself. This was achieved by grouping multiple shots together. From internal testing (results not shown), it was found that grouping four shots together into a single segment improved the reconstruction performance, whereas still providing adequate temporal resolution (4 s, with the number of motion parameters reducing from 392 to 98). Regarding the set of spatially low‐order basis functions, the solid harmonics (SH) were used following Ref 16. Whereas Ref. 16 used second‐order SH, we tested performance for orders 1 to 3 and found first‐order SH to perform optimally. Due to higher noise levels compared to high‐field MRI, the proposed outlier rejection in Ref. 12 did not prove effective. Because outlier rejection excludes data during the image reconstruction, it decreases the available SNR. Given its ineffectiveness in this study, combined with the already limited available SNR, the outlier rejection was not used in this work.

Reconstructions are performed on a 20(40) × Intel Xeon Silver 4210 2.20 GHz CPU, 251 GB RAM, 32GB Nvidia Tesla V100 GPU (Santa Clara, CA). The maximum computation time for a motion‐corrected reconstruction was 2 min.

### 3.4 Image processing

To enhance the SNR of the acquired images, the magnitude of both echoes is averaged. Because resulting pairs of images have different spatial distortions, they were first registered to each other using an affine transformation.^21^ The averaged image will be referred to as the *combined image*.

### 3.5 Image evaluation

Given that no ground truth is available, the proposed reconstruction methods are evaluated by comparing pairs of images using the different reconstruction methods. Each pair of images is first spatially aligned using rigid body registration; then, a brain mask is applied and the normalized mean squared error (NMSE) computed between them. This builds upon the assumption that if there were no remaining errors from either motion/phase changes or other nonidealities, acquisitions of the same subject with the same contrast would have the same anatomical content. Such images would only differ by noise, which would be uncorrelated between the two acquisitions being compared, and this constitutes the minimal possible NMSE. Any other image differences, specifically those resulting from imperfect correction, would increase the NMSE. The use of brain masking removes potentially pose‐dependent incidental changes in extracerebral areas. Image registration was performed using a rigid motion model. Brain masks were created for each subject by manual delineation. Two distinct image pairs have been considered: (A) minimal motion and the deliberate motion 1, and (B) deliberate motion 1 and deliberate motion 2.

## 4 RESULTS

**FIGURE 2:**
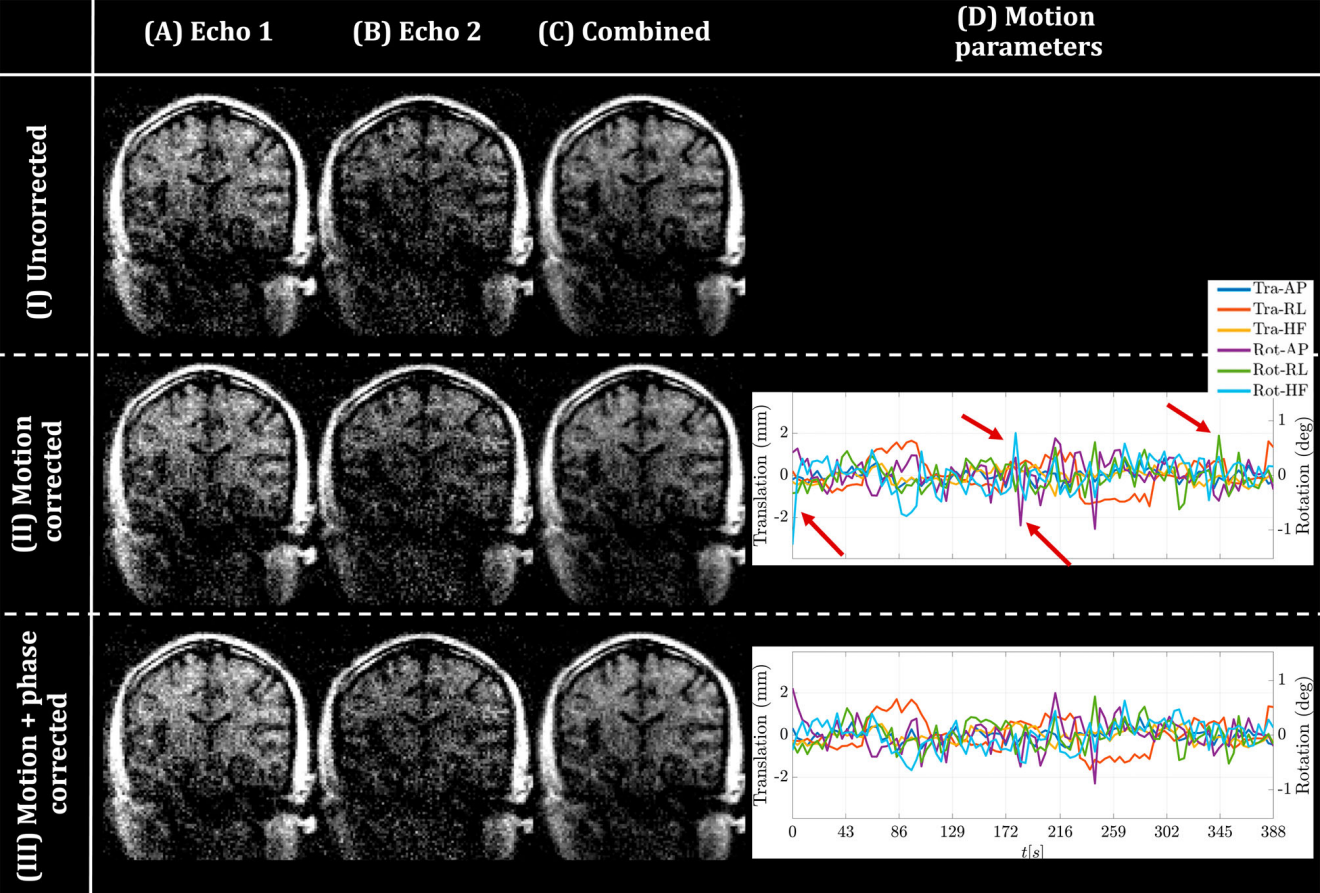
Different reconstruction methods (rows) are shown for the individual and combined echoes (A–C) for the deliberate motion 1 experiment in HV2. (D) The motion parameters of the first echo are shown on the right, where suspected outliers are annotated with red arrows. HV, healthy volunteer.

Figure 2 shows the three different reconstruction methods on a single acquisition (rows I–III) applied to both echoes (columns A and B). The combined image is shown in column C. Column D shows time‐resolved motion estimates for both correction techniques. For both echoes, as well as the combined image, the motion correction (II) shows a reduction of motion artifacts, with further (more subtle) improvements with the motion + phase correction (III). The proposed phase correction also results in more orderly motion estimates (D), with fewer potential outliers observed in the motion estimates compared to a motion‐only correction (red arrows).

**FIGURE 3:**
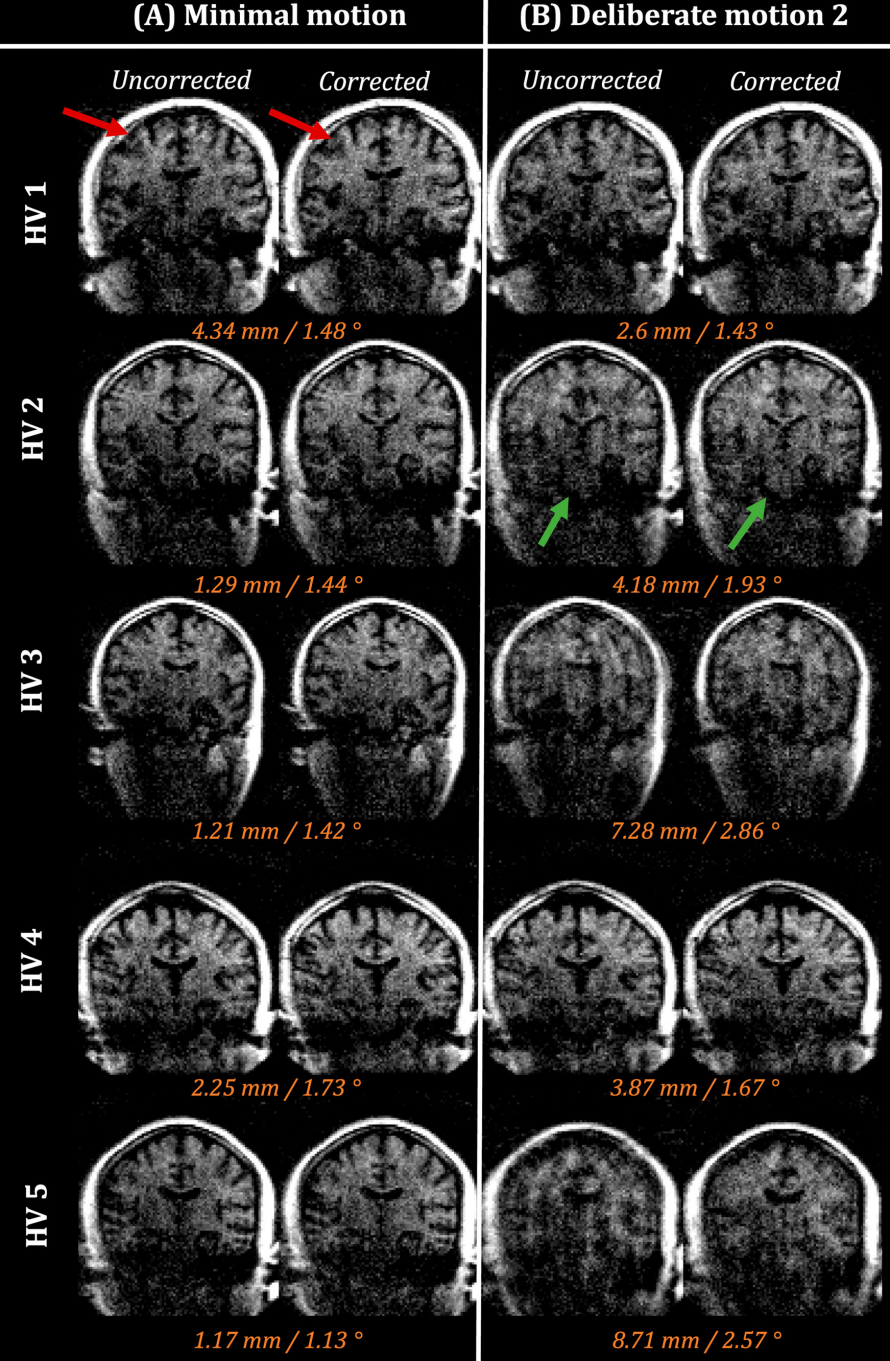
Uncorrected and proposed corrected reconstructions are shown for the (A) minimal motion and (B) deliberate motion 2 experiment. Reconstructions are shown for all the HV (rows). As a guidance of the amount of motion present, the range of estimated translation and rotation are shown in orange for each experiment.

Due to the improved performance of the proposed motion + phase correction, only this method will be shown from this point onward when referring to the corrected images. Figure 3 shows the uncorrected and corrected images for all HVs^1^, ^2^, ^3^, ^4^, ^5^ for both the minimal motion and deliberate motion 2 experiment (columns A and B, respectively). Ranges (maximum–minimum) for both translation and rotation parameters are displayed in orange to indicate the extent of estimated motion in each experiment for each HV. For the minimal motion experiment, the improvements are more subtle, except for HV1, where clear improvements can be observed. For the deliberate motion 2 experiment, consistent improvements in image quality are observed across most HVs. Improvements appear as both reductions in motion artifacts (red arrow), as well as signal recovery in certain areas in the brain (green arrows). It is noteworthy that the estimated levels of motion differ a lot between HVs, resulting in different levels of motion artifacts. In the most extreme case (HV 5, column B), the motion artifacts could not be fully resolved.

**FIGURE 4:**
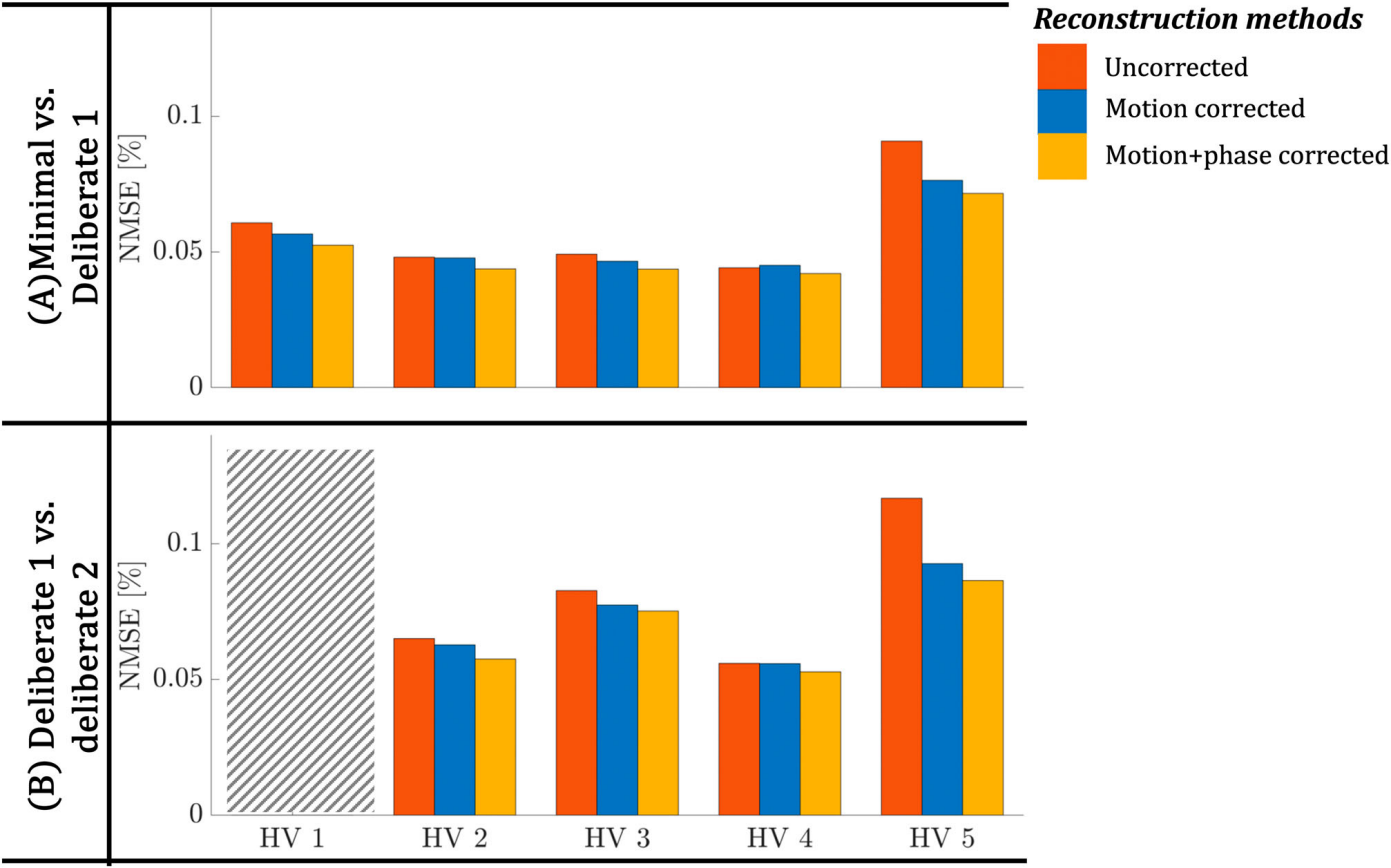
The NMSEs are shown for both comparisons. (A, B) Minimal vs. deliberate and deliberate vs. deliberate experiment. NMSEs are shown for all HVs (columns) and reconstruction methods (colors). Decreased NMSE reflects improved reconstruction consistency. Mean values across HVs are 0.059, 0.055, and 0.051 for the uncorrected, motion correction, and motion + phase correction, respectively. NMSE, normalized mean‐squared‐errors.

Quantitative image evaluation between the two tested acquisition pairings is shown in Figure 4 for all HVs (columns) and types of reconstruction (colors). For four out of five HVs, the motion‐only correction yields better performance than a simple SENSE reconstruction alone, with consistent further improvements from the proposed motion + phase correction. Note that the latter achieves the best performance for all HVs.

**FIGURE 5:**
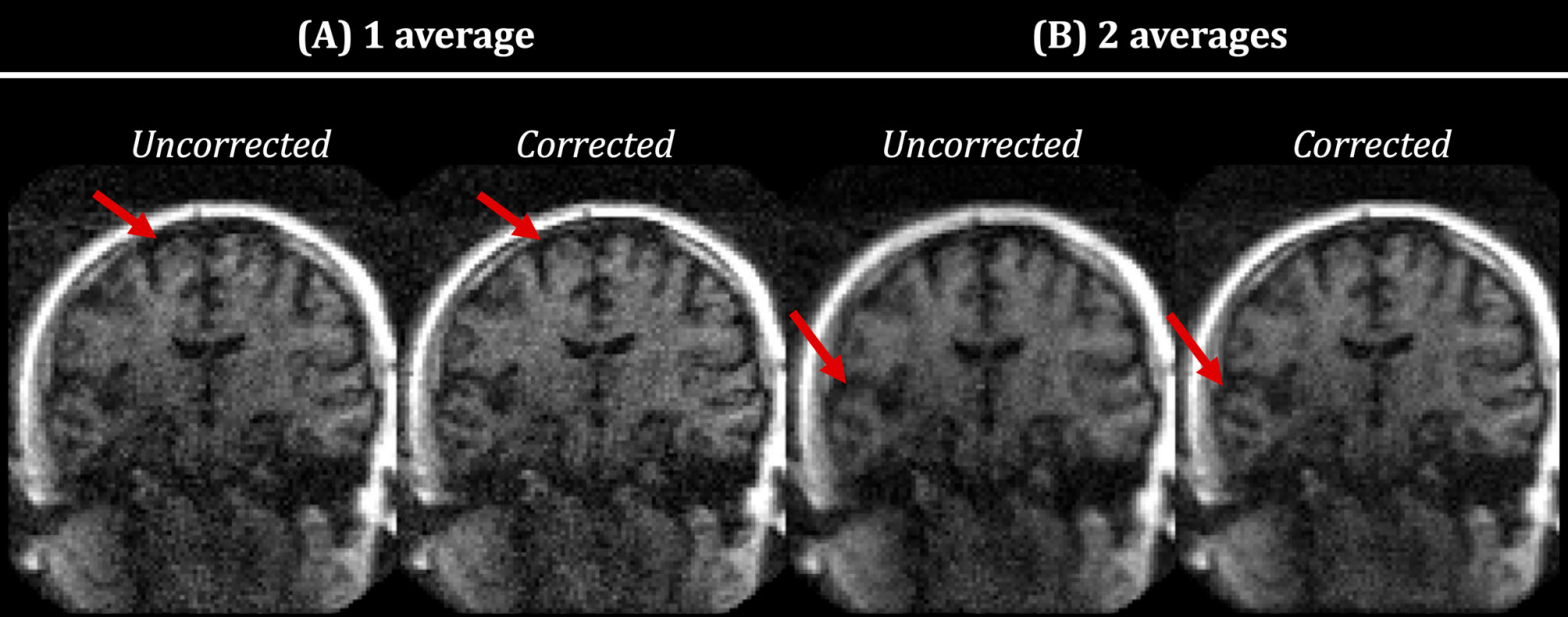
Uncorrected and the proposed corrected reconstructions in HV1 for acquisitions consisting of (A) 1 and (B) 2 averages, corresponding to acquisition times of 6 min 32 s and 13 min 4 s, respectively.

Finally, Figure 5 compares the reconstructed images for HV1 when using (A) a single acquisition versus (B) two averages. As expected, increasing the amount of acquired data improves the SNR, whereas the proposed correction can still correct for motion and phase variations.

## 5 DISCUSSION

This study demonstrates the feasibility of applying motion correction to an ULF MRI system for brain imaging. By leveraging advanced methods such as alignedSENSE and incorporating phase‐informed corrections, this research highlights the potential to improve image quality even in the presence of minimal or involuntary patient motion.

Results in Figure 2 show that the motion correction can be successfully applied to individual echo images. The additional correction of between‐shot phase variations resulted in more coherent motion traces, accompanied by further improvement in image quality. Figure 3 confirms that these observations apply for all the HVs tested and all motion experiments. Visual improvements are supported by quantitative results shown in Figure 4. Finally, Figure 5 shows the flexibility to apply this framework when combining multiple averages to increase the SNR while still enabling motion correction.

Motion‐corrected reconstruction may be particularly relevant for ULF MRI systems, where the inherent limitations in SNR demand longer acquisition times, which in turn increase the likelihood of subject motion. The ability to mitigate any resulting artifacts could broaden the clinical applicability of portable ULF MRI systems, making them a more viable option for point‐of‐care imaging in diverse healthcare settings. Despite some cases in which extreme motion could not be fully corrected, the overall performance of the proposed method is promising, with improved reconstructions in all cases.

A notable difference between ULF and higher field images, which the data presented here illustrate, is that the distinctive ghosting artifacts resulting from patient motion can go largely unnoticed given the low intrinsic SNR of the imaging data. Nevertheless, signal loss due to motion can still occur. As a result, certain cases of motion artifacts might be incorrectly perceived as an apparent loss of available SNR (e.g., HV2 in Figure 3). It is possible that the ULF images that appear to have variable SNR between acquisitions are in fact damaged by undiagnosed motion corruption. It is noteworthy that the corrected images still have limited SNR, and that longer scanning is needed to get desirable SNR properties. As shown for the preliminary data in Figure 5, this prolonged scanning is still compatible with the proposed methods in this paper.

Although our initial results show promising results for motion correction at ULF, further testing will be needed to determine the implications for image interpretation and analysis in a clinical setting. In this first study, only one echo train length was tested, with only one resolution and tiling pattern. There may well be scope to optimize further, and different settings may be valuable for different scenarios. Also, more subtle effects due to the tile‐reordering, such as on imaging point spread function, need to be explored. Robustness against these design choices, such as shot grouping, should be characterized and evaluated. Furthermore, a wider range of state‐of‐the‐art motion‐correction techniques may need to be assessed to understand the overall potential of motion correction at ULF.^9^ Scout‐based methods (e.g., scout accelerated motion estimation and reduction) are of especial interest because they could potentially achieve higher SNR reconstructions by leveraging scout information into the reconstruction algorithm. However, scout‐based methods rely on rapid acquisition of an image to estimate motion, which might be challenging at the low SNR at ULF. Additionally, regularized reconstruction methods should be explored in combination with motion‐correction algorithms. Deep learning models already tested in the setting of ULF imaging are likely to combine well with motion correction.^22^, ^23^ Finally, the ability to correct motion should be explored for variable density sampling patterns, which are actually relevant, or for ULF MRI given the limited intrinsic SNR.

## 6 CONCLUSION

This study explores the feasibility of applying motion‐correction techniques to ULF MRI systems. We performed a small pilot study in 5 HV that showcased successful motion correction for ULF acquisitions, which are likely to be longer in duration than high(er) field MRI to achieve the needed SNR.

## FUNDING INFORMATION

Funding from the Bill & Melinda Gates Foundation. Also from The National Institute for Health and Care Research (NIHR) Maudsley Biomedical Research Centre (BRC) at the South London and Maudsley NHS Foundation Trust.

Core funding was provided by the Wellcome/EPSRC Centre for Medical Engineering [WT203148/Z/16/Z].

T.A. was supported by a Medical Reserve Corps (MRC) Senior Clinical Fellowship [MR/Y009665/1]; and the MRC Centre for Neurodevelopmental Disorders, King's College London [MR/N026063/1].

## CONFLICT OF INTEREST STATEMENT

Rui Teixeira is currently employed by Hyperfine Inc., and Seon Deoni is currently employed by the Bill & Melinda Gates Foundation.

## Supporting information


**FIGURE S1.** Example of the coil profiles for the HyperFine system. Different coil elements are shown across the columns.
**FIGURE S2.** Motion traces for all HVs for the “deliberate motion 1” experiment.
