# Supplementary material for: Motion‐corrected brain MRI at ultralow field (64 mT)
Source: Magn Reson Med. 2025 Mar 28;94(2):825–34. doi: 10.1002/mrm.30506 (PMC12137760; doi:10.1002/mrm.30506)

## Supporting Information:

**Figure S1:** Example of the coil profiles for the HyperFine system. Different coil elements are shown across the columns.


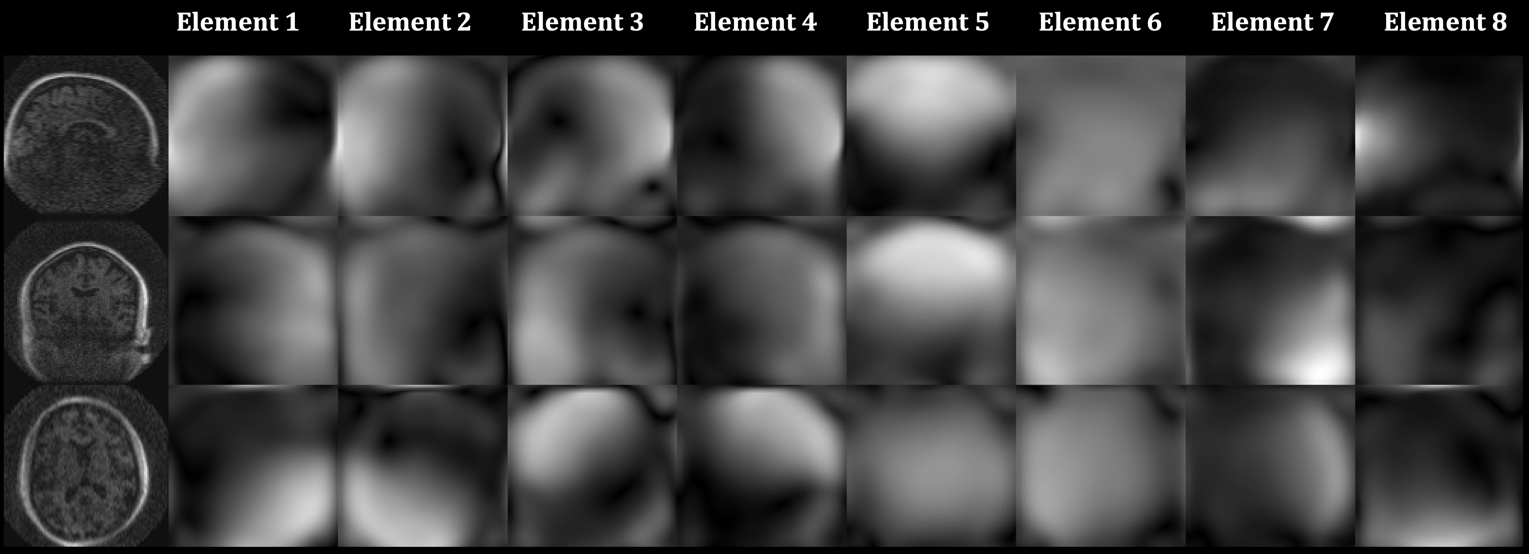


**Figure S2:** Motion traces for all HVs for the “deliberate motion 1” experiment.


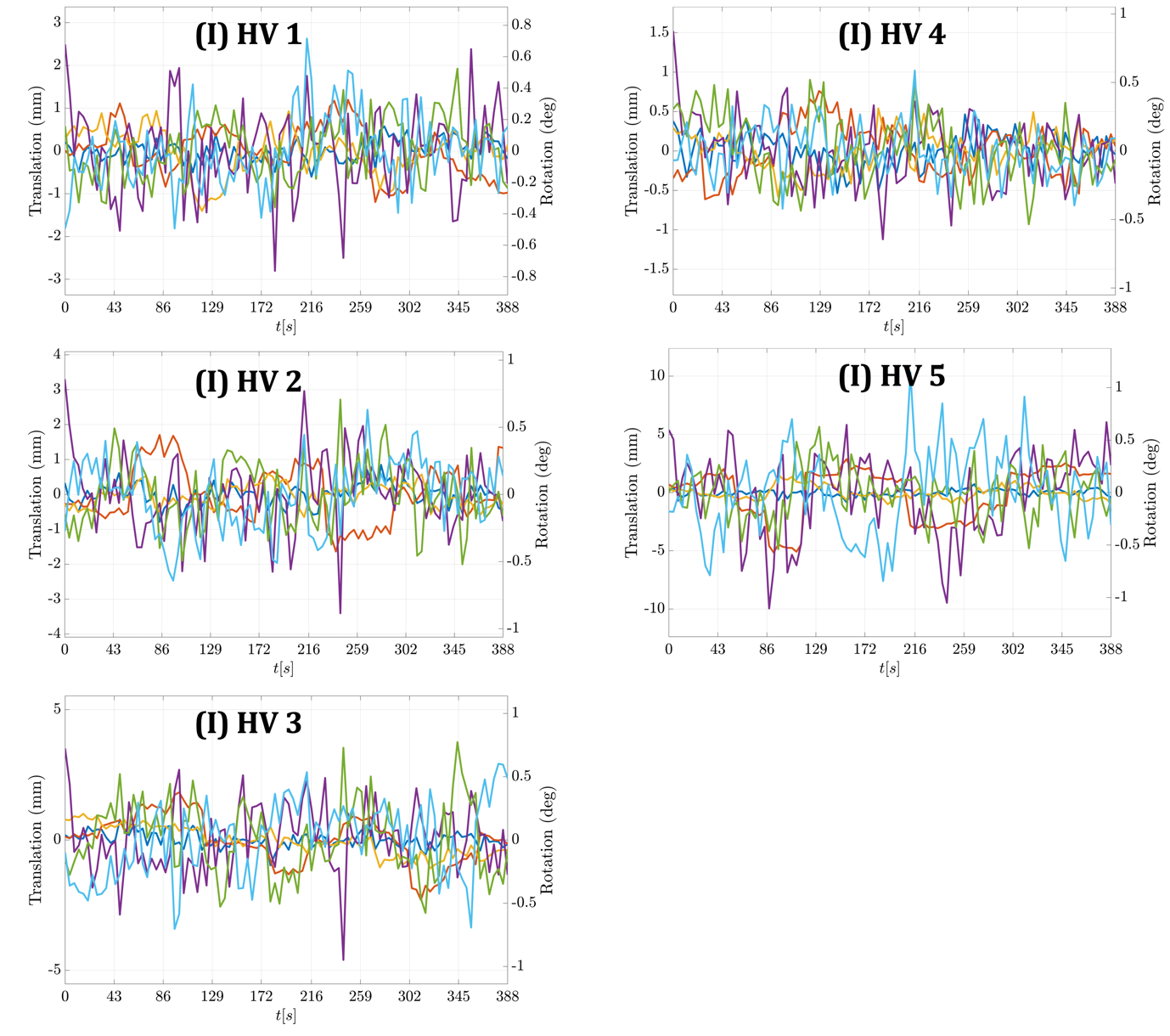

Supplement: Supplementary file 1 — FIGURE S1. Example of the coil profiles for the HyperFine system. Different coil elements are shown across the columns. FIGURE S2. Motion traces for all HVs for the “deliberate motion 1” experiment. [file MRM-94-825-s001.docx]
